# Apolipoprotein A-IV and its derived peptide, T55−121, improve glycemic control and increase energy expenditure

**DOI:** 10.1093/lifemeta/loae010

**Published:** 2024-03-14

**Authors:** Zhen Cao, Lei Lei, Ziyun Zhou, Shimeng Xu, Linlin Wang, Weikang Gong, Qi Zhang, Bin Pan, Gaoxin Zhang, Quan Yuan, Liujuan Cui, Min Zheng, Tao Xu, You Wang, Shuyan Zhang, Pingsheng Liu

**Affiliations:** National Laboratory of Biomacromolecules, CAS Center for Excellence in Biomacromolecules, Institute of Biophysics, Chinese Academy of Sciences, Beijing 100101, China; University of Chinese Academy of Sciences, Beijing 100049, China; National Laboratory of Biomacromolecules, CAS Center for Excellence in Biomacromolecules, Institute of Biophysics, Chinese Academy of Sciences, Beijing 100101, China; The State Key Laboratory of Bioactive Substance and Function of Natural Medicines, Institute of Materia Medica, Chinese Academy of Medical Sciences and Peking Union Medical College, Beijing 100050, China; Luxembourg Centre for Systems Biomedicine, University of Luxembourg, Esch-sur-Alzette 4362, Luxembourg; National Laboratory of Biomacromolecules, CAS Center for Excellence in Biomacromolecules, Institute of Biophysics, Chinese Academy of Sciences, Beijing 100101, China; Guangzhou Regenerative Medicine and Health Guangdong Laboratory (Bioland Laboratory), Guangzhou, Guangdong 510005, China; Department of Computer Science, School of Computing, National University of Singapore, Singapore 117417, Singapore; National Laboratory of Biomacromolecules, CAS Center for Excellence in Biomacromolecules, Institute of Biophysics, Chinese Academy of Sciences, Beijing 100101, China; University of Chinese Academy of Sciences, Beijing 100049, China; National Laboratory of Biomacromolecules, CAS Center for Excellence in Biomacromolecules, Institute of Biophysics, Chinese Academy of Sciences, Beijing 100101, China; University of Chinese Academy of Sciences, Beijing 100049, China; School of Basic Medical Sciences, Southwest Medical University, Luzhou, Sichuan 646000, China; School of Basic Medical Sciences, Southwest Medical University, Luzhou, Sichuan 646000, China; National Laboratory of Biomacromolecules, CAS Center for Excellence in Biomacromolecules, Institute of Biophysics, Chinese Academy of Sciences, Beijing 100101, China; The State Key Laboratory for Diagnosis and Treatment of Infectious Diseases, National Clinical Research Center for Infectious Diseases, Collaborative Innovation Center for Diagnosis and Treatment of Infectious Diseases, The First Affiliated Hospital, College of Medicine, Zhejiang University, Hangzhou, Zhejiang 310003, China; National Laboratory of Biomacromolecules, CAS Center for Excellence in Biomacromolecules, Institute of Biophysics, Chinese Academy of Sciences, Beijing 100101, China; University of Chinese Academy of Sciences, Beijing 100049, China; Guangzhou Regenerative Medicine and Health Guangdong Laboratory (Bioland Laboratory), Guangzhou, Guangdong 510005, China; National Laboratory of Biomacromolecules, CAS Center for Excellence in Biomacromolecules, Institute of Biophysics, Chinese Academy of Sciences, Beijing 100101, China; Beijing Key Laboratory of Emerging Infectious Diseases, Institute of Infectious Diseases, Beijing Ditan Hospital, Capital Medical University, Beijing 100015, China; Beijing Institute of Infectious Diseases, Beijing 100015, China; National Center for Infectious Diseases, Beijing Ditan Hospital, Capital Medical University, Beijing 100015, China; National Key Laboratory of Intelligent Tracking and Forecasting for Infectious Diseases, Beijing 100015, China; National Laboratory of Biomacromolecules, CAS Center for Excellence in Biomacromolecules, Institute of Biophysics, Chinese Academy of Sciences, Beijing 100101, China; University of Chinese Academy of Sciences, Beijing 100049, China

**Keywords:** bariatric/metabolic surgeries, proteomics, apolipoprotein A-IV, glucose tolerance, glucose-stimulated insulin secretion, human islets

## Abstract

It is crucial to understand the glucose control within our bodies. Bariatric/metabolic surgeries, including laparoscopic sleeve gastrectomy (LSG) and Roux-en-Y gastric bypass (RYGB), provide an avenue for exploring the potential key factors involved in maintaining glucose homeostasis since these surgeries have shown promising results in improving glycemic control among patients with severe type 2 diabetes (T2D). For the first time, a markedly altered population of serum proteins in patients after LSG was discovered and analyzed through proteomics. Apolipoprotein A-IV (apoA-IV) was revealed to be increased dramatically in diabetic obese patients following LSG, and a similar effect was observed in patients after RYGB surgery. Moreover, recombinant apoA-IV protein treatment was proven to enhance insulin secretion in isolated human islets. These results showed that apoA-IV may play a crucial role in glycemic control in humans, potentially through enhancing insulin secretion in human islets. ApoA-IV was further shown to enhance energy expenditure and improve glucose tolerance in diabetic rodents, through stimulating glucose-dependent insulin secretion in pancreatic β cells, partially via Gαs-coupled GPCR/cAMP (G protein-coupled receptor/cyclic adenosine monophosphate) signaling. Furthermore, T55−121, truncated peptide 55−121 of apoA-IV, was discovered to mediate the function of apoA-IV. These collective findings contribute to our understanding of the relationship between apoA-IV and glycemic control, highlighting its potential as a biomarker or therapeutic target in managing and improving glucose regulation.

## Introduction

Over the past decades, there has been a striking surge in the global occurrence of obesity and type 2 diabetes (T2D), leading to significant social and economic consequences [1]. Bariatric surgery has been utilized as a means of weight reduction for individuals diagnosed with morbid obesity as well as an effective intervention that can lead to significant improvements in diabetes management [2]. Currently, laparoscopic sleeve gastrectomy (LSG) and laparoscopic Roux-en-Y gastric bypass (RYGB) are the two most commonly performed procedures in the field of bariatric surgery. Both LSG and RYGB have been found to yield enduring weight loss, long-term improvement in glycemic control, and other notable metabolic improvements in patients with morbid obesity and severe T2D [3, 4]. Exploring the significant altered factors associated with substantial improvement of T2D after surgery would aid in understanding the mechanisms underlying glucose control.

Apolipoprotein A-IV (apoA-IV) is almost exclusively synthesized by the enterocytes of the small intestine, with dietary lipids serving as significant stimulants for its production [5, 6]. Of note, the production of apoA-IV accounts for 3% of total protein synthesis following oleic acid infusion [7, 8]. The protein exists in blood circulation as both lipoproteins (chylomicron and high-density lipoprotein) and in a lipoprotein-free form after the metabolism of chylomicrons [9]. Adiposome-derived artificial lipoproteins bearing apoA-IV are established and exhibit functions in the improvement of glucose tolerance [10]. Importantly, apoA-IV is shown to rise after RYGB in humans, and simultaneously, there is a noticeable improvement in diabetes [11, 12]. Additionally, population-based genotype studies showed that apoA-IV polymorphism, especially apoA-IV^360^, has a significant effect on serum glucose levels [13, 14]. These findings indicate that apoA-IV plays a role in the regulation of glucose levels.

Further, Wang *et al*. reported that apoA-IV augments glucose-stimulated insulin secretion (GSIS) in wild-type (WT) mice [15]. In addition, Li *et al*. found that apoA-IV reduces hepatic gluconeogenesis through interaction with nuclear receptor NR4A1 (nuclear receptor subfamily 4 group A member 1) and NR1D1 (nuclear receptor subfamily 1 group D member 1, also known as REV-ERBα) to repress phosphoenolpyruvate carboxykinase (PEPCK) and glucose-6-phosphatase (Glc-6-Pase) expression at the transcriptional level [16, 17]. Li *et al*. also found that apoA-IV contributes to improved insulin sensitivity in adipocytes via phosphatidylinositol 3 kinase (PI3K)-protein kinase B (AKT) signaling [18]. However, it is unknown whether apoA-IV enhances insulin secretion in humans and the mechanism by which apoA-IV enhances insulin secretion is not fully understood. Furthermore, it is crucial to identify the specific functional peptide of apoA-IV that plays a role in modulating glucose homeostasis, which would potentially offer therapeutic benefits in managing obesity and T2D.

Here, for the first time, the patient serum proteins were compared before and after LSG through gel-based proteomics to reveal the potential proteins involved in glycemic control in humans. In the most changed bands, apoA-IV was shown to be the most abundant protein and was revealed to increase extremely after LSG. Besides, we also found that apoA-IV was increased significantly in patients following RYGB. All the data implied that apoA-IV plays a role in glucose control in humans. Further, we found that administrating eukaryote-derived recombinant apoA-IV resulted in an enhanced energy expenditure and improved glucose tolerance in both WT and diabetic rodents. Furthermore, our findings showed that apoA-IV stimulated GSIS in human islets, partially via Gαs-coupled GPCR/cAMP (G protein-coupled receptor/cyclic adenosine monophosphate) signaling. Moreover, we identified that truncated peptide 55−121 (T55−121) of murine apoA-IV improved glucose tolerance and enhanced metabolic rate. These findings would expand our knowledge of the relationship between apoA-IV and glycemic control, underscoring its potential as a biomarker or therapeutic target for enhancing glucose regulation.

## Results

### ApoA-IV rises in sera of patients after bariatric surgeries

Bariatric surgeries, such as LSG and RYGB, have been identified as effective procedures for long-term enhancement in glycemic control and other significant metabolic improvements in patients suffering from morbid obesity and T2D. To gain a better understanding of the underlying mechanisms involved in glucose control, it is essential to identify the significant factors that undergo noticeable changes following bariatric surgeries. Thus in this study, we performed analysis of patient sera before and after undergoing either LSG or RYGB procedures.

**Figure 1:**
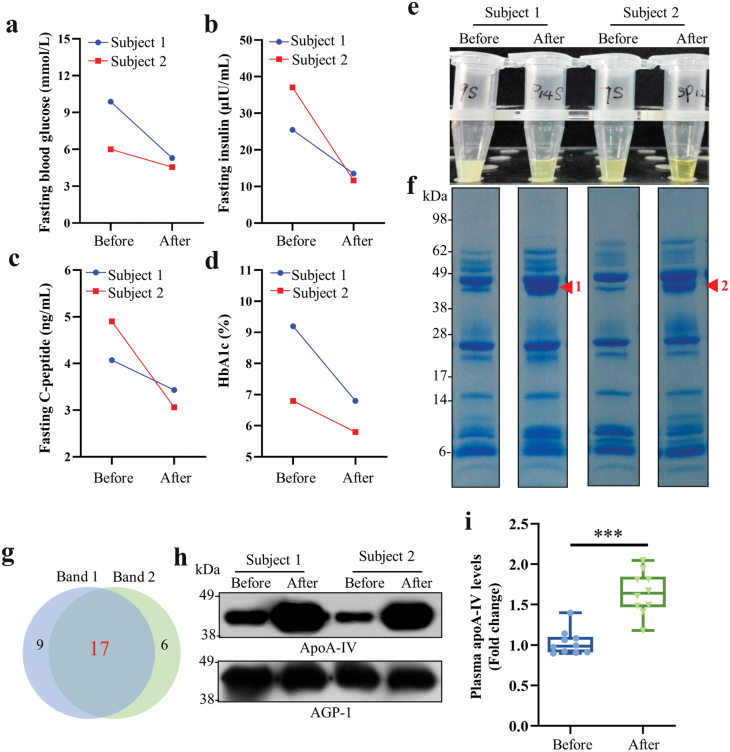
The levels of apoA-IV increase in the sera of patients after bariatric surgeries. (a–d) Changes in serum biochemical parameters of patients with morbid obesity and T2D before and one year after LSG, including fasting blood glucose (a), fasting insulin (b), fasting C-peptide (c), and HbA1c (d). (e and f) Protein profiles in sera collected from patients before and after LSG (e) analyzed by SDS-PAGE (f). Protein bands 1 and 2 from post-surgery samples, indicated by red arrowheads, were subjected to MS-based proteomics. (g and h) Identification of proteins in bands 1 and 2 based on proteomics and immunoblot analysis. Proteomic analysis of bands 1 and 2 (g). Immunoblot analysis of apoA-IV and AGP-1 in sera of subjects before and after LSG (h). AGP-1 is a loading control. (i) The changes of plasma apoA-IV levels of diabetic obese patients (*n* = 10) before and one year after RYGB were analyzed based on the reported proteomics data [19]. Data are presented as mean ± SEM. Statistical significance was determined by the two-tailed Student’s *t*-test. ^***^P < 0.001.

Similar to previous reports [3], patients showed significant improvement in glycemic control one year after LSG, as evidenced by a substantial reduction in fasting blood glucose, insulin, C-peptide, and hemoglobin A1c (HbA1c) levels (Fig. 1a−d). Compared with sera before LSG, the post-surgery sera exhibited a noticeable reduction in turbidity (Fig. 1e). To investigate the significant factors that undergo notable changes in patient sera after LSG, for the first time, we performed a comparative analysis of serum proteins before and after LSG through gel-based proteomics. Notably, similar protein bands, 1 and 2, around 45 kDa in SDS-PAGE, were remarkably increased in both patient sera after LSG (indicated as red arrowhead in Fig. 1f), the proteins in which could potentially play crucial roles in the improvement of glycemic control. Then both bands were subjected to proteomic analysis using mass spectrometry (MS) to identify the major proteins. There were 26 proteins identified in band 1 (Supplementary Table S1) and 23 proteins in band 2 (Supplementary Table S2) with 17 proteins in common (Fig. 1g). Among the 17 common proteins, apoA-IV achieved the highest ranking with the highest score, coverage, and the largest number of unique peptide segments. More importantly, apoA-IV level in serum was revealed to be elevated substantially after LSG in both patients, through immunoblotting, using alpha-1-acid glycoprotein (AGP-1), one of the 17 common proteins, as a loading control (Fig. 1h).

Besides the effects of LSG, we conducted an analysis of the factors that experienced changes in the plasma of patients before and one year after RYGB as well. The analysis was performed using the reported proteomics data [19]. One year after RYGB, the fasting blood glucose levels of the 10 diabetic obese patients exhibited a notable decrease, transitioning from levels higher than 9 mmol/L to levels lower than 7 mmol/L [19]. Moreover, consistent with the effect of LSG, plasma apoA-IV level demonstrated a remarkable increase one year after RYGB surgery (Fig. 1i; Suppplementary Table S3).

Collectively, these data consistently demonstrated a significant increase in apoA-IV levels in patients, accompanied by long-term enhancement in glycemic control. Therefore, apoA-IV may be important in glycemic control in humans.

### ApoA-IV improves glucose tolerance in rodents

**Figure 2:**
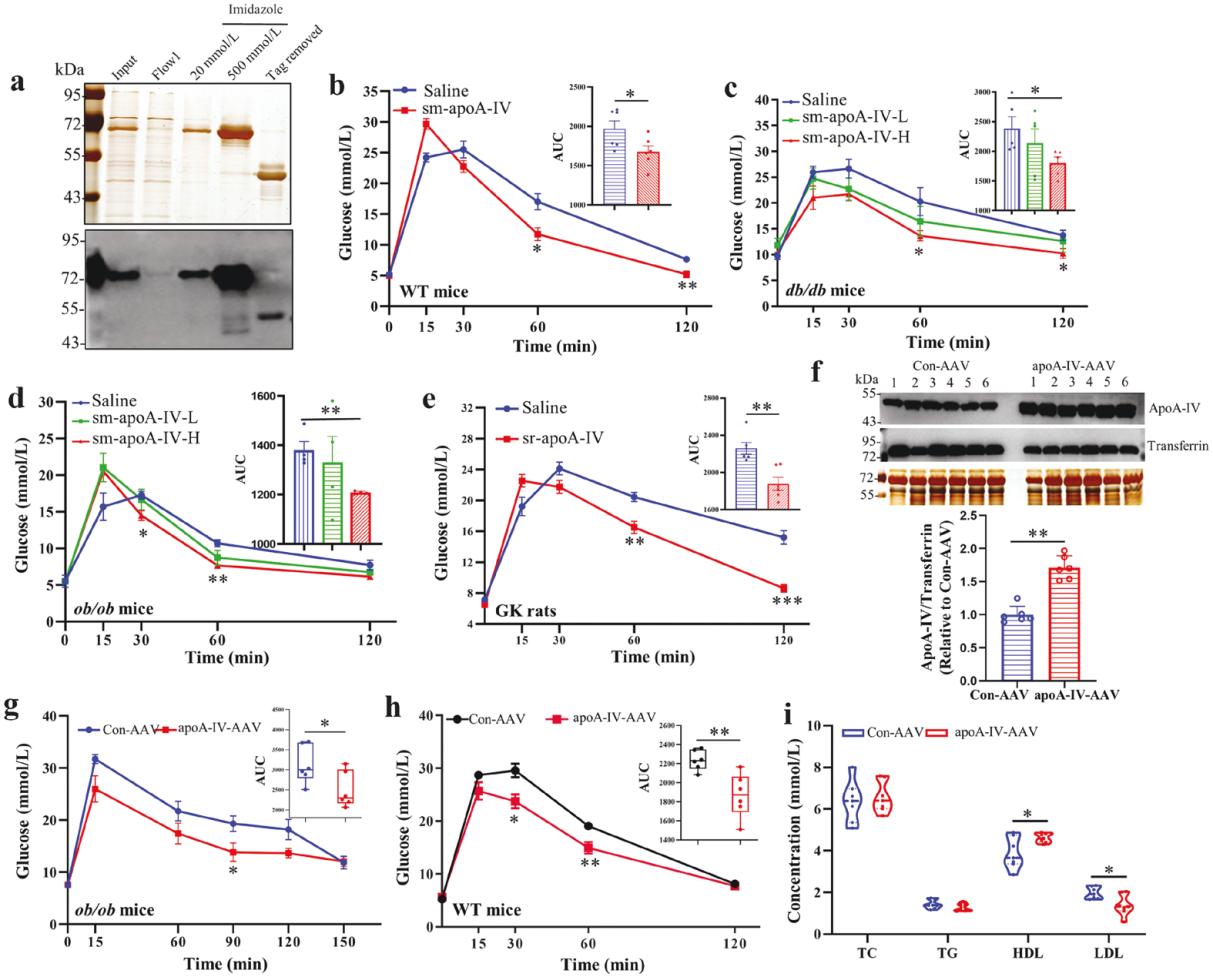
Administration of apoA-IV and overexpression of apoA-IV improve glucose tolerance in rodents. (a) Silver staining (top) and immunoblot analysis (bottom) of recombinant apoA-IV protein purified from a eukaryotic expression system (HEK293F cells). (b) The IPGTT in WT mice (*n* = 6) with saline or sm-apoA-IV (6 mg/kg body weight) treatment. (c) The IPGTT in *db/db* mice (*n* = 5) with saline, sm-apoA-IV-L (3 mg/kg body weight), or sm-apoA-IV-H (6 mg/kg body weight) treatment. (d) The IPGTT in *ob/ob* mice (*n* = 4) with saline, sm-apoA-IV-L (3 mg/kg body weight), or sm-apoA-IV-H (6 mg/kg body weight) treatment. (e) The IPGTT in GK rats (*n* = 6) with saline or sr-apoA-IV treatment (6 mg/kg body weight) treatment. (f) Immunoblot analysis of apoA-IV (top) in sera from *ob/ob* mice infected with Con-AAV or apoA-IV-AAV, with transferrin as a loading control, and quantification of serum apoA-IV levels in both groups (bottom). (g) The IPGTT in *ob/ob* mice infected with Con-AAV or apoA-IV-AAV (*n* = 6). (h) The IPGTT in WT mice infected with Con-AAV or apoA-IV-AAV (*n* = 6). (i) Serum biochemical changes of AAV-infected *ob/ob* mice. Data are presented as mean ± SEM. Statistical significance was determined by the two-tailed Student’s *t*-test. ^*^P < 0.05, ^**^P < 0.01. AUC is the area under the curve. sm-apoA-IV, signal peptide-removed mouse apoA-IV. sr-apoA-IV, signal peptide-removed rat apoA-IV. Con-AAV, adeno-associated virus encoding GFP. apoA-IV-AAV, adeno-associated virus encoding apoA-IV.

To investigate the potential involvement of apoA-IV in improving glucose homeostasis, we administered exogenous apoA-IV protein directly to both WT and T2D rodents via intraperitoneal injection. Recombinant apoA-IV protein was expressed in HEK293 cells and purified for use (Fig. 2a). The administration of apoA-IV significantly improved the glucose tolerance in WT mice (Fig. 2b). Then we tested whether apoA-IV had a beneficial effect on glucose tolerance in T2D rodents. The results showed that the eukaryote-derived apoA-IV treatment led to improved glucose tolerance in both *db/db* mice and *ob/ob* mice in a dose-dependent manner (Fig. 2c and d). Similarly, glucose tolerance was enhanced in apoA-IV-treated Goto-Kakizaki (GK) rats (Fig. 2e).

In addition to studying the effect of apoA-IV through administration, we also investigated its physiological effects in both WT and diabetic mice by overexpressing apoA-IV *in vivo* using an adeno-associated virus (AAV). After 1 month of apoA-IV-AAV infection, serum apoA-IV level was 70% higher than that observed in control-AAV-treated mice (Fig. 2f). The expression of exogenous apoA-IV was also detected in tissues, and the results indicated the presence of apoA-IV-Flag in the small intestine, pancreas, and liver (Supplementary Fig. S1). Although no differences were evident in body weight between the two groups (Supplementary Fig. S2), *ob/ob* mice overexpressing apoA-IV exhibited improved glucose tolerance compared to the control mice (Fig. 2g). Similarly, apoA-IV overexpression also enhanced the glucose tolerance of WT mice (Fig. 2h). Notably, *ob/ob* mice overexpressing apoA-IV exhibited elevated levels of high-density lipoprotein (HDL) and lower levels of low-density lipoprotein (LDL) (Fig. 2i), suggesting that apoA-IV overexpression is beneficial for maintaining cholesterol homeostasis. Indeed, previous research has shown that apoA-IV may protect against atherosclerosis [20] and cAMP-stimulated cholesterol efflux from macrophages is induced in transgenic mice overexpressing human apoA-IV [21].

Taken together, these results demonstrated that both administration of recombinant apoA-IV protein and overexpression of apoA-IV *in vivo* using AAV improve glucose tolerance in both WT and diabetic rodents.

### ApoA-IV enhances insulin secretion from human and rodent islets, partially via Gαs-coupled GPCR/cAMP signaling

**Figure 3:**
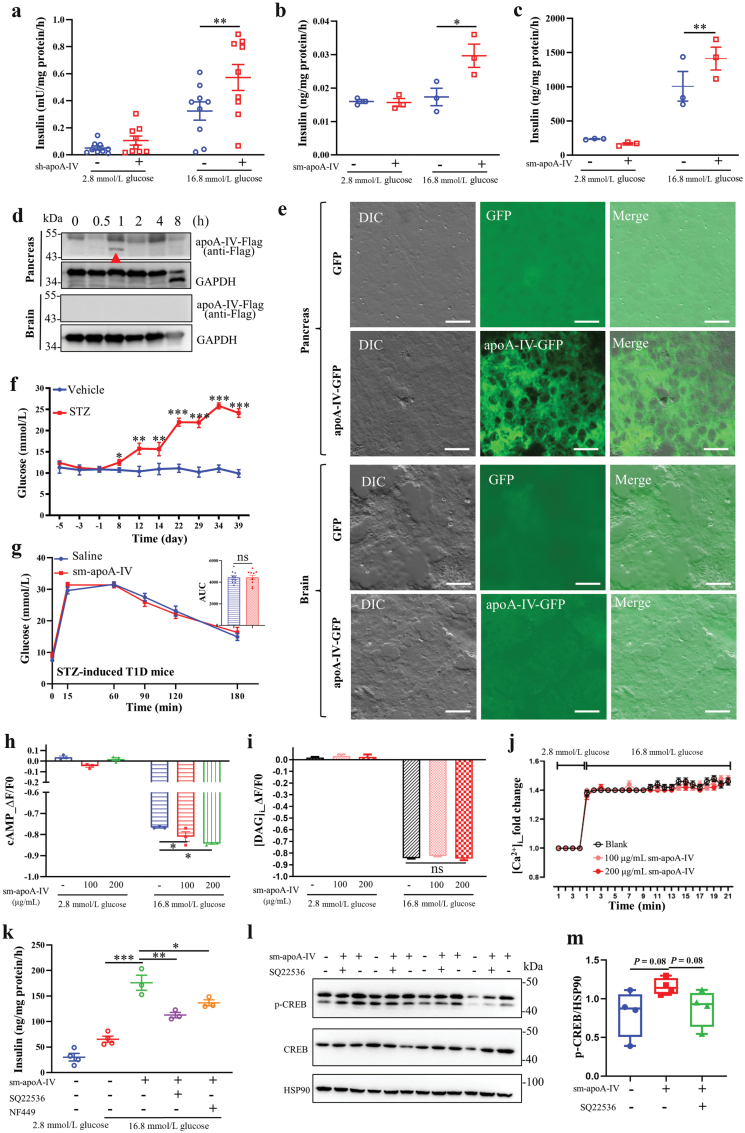
ApoA-IV promotes GSIS in human primary islets, partially through Gαs-coupled GPCR/cAMP signaling. (a) GSIS in human primary islets treated with vehicle or 100 μg/mL sh-apoA-IV for 3 h. (b) GSIS in spontaneous T2D KKAy mouse primary islets treated with vehicle or 100 μg/mL sm-apoA-IV for 3 h. (c) GSIS assay in MIN6 cells treated with vehicle or 100 μg/mL sm-apoA-IV for 3 h. (d) Immunoblot analysis of apoA-IV-Flag in pancreas (top) and brain (bottom) at indicated times after intraperitoneal administration of recombinant apoA-IV-Flag protein. GAPDH is a loading control. (e) Fluorescent sections of pancreas and brain after 2 h incubation with 10 μg/mL apoA-IV-GFP or GFP. Scale bar = 20 μm. (f) Blood glucose levels in mice with vehicle or STZ treatment. (g) The IPGTT in STZ-induced T1D mice (*n* = 10) with the administration of saline or sm-apoA-IV (6 mg/kg body weight). (h) The cAMP sensor fluorescence changes in MIN6 cells with 0, 100, or 200 μg/mL sm-apoA-IV. (i) Fluorescent DAG sensor changes in MIN6 cells treated with 0, 100, or 200 μg/mL sm-apoA-IV. (j) Fluorescent Ca^2+^ sensor changes in MIN6 cells treated with 0, 100, or 200 μg/mL sm-apoA-IV. (k) GSIS in MIN6 cells treated with 100 μg/mL sm-apoA-IV alone, 100 μg/mL sm-apoA-IV together with 20 μmol/L SQ22536 (AC inhibitor), or 100 μg/mL sm-apoA-IV together with 10 μmol/L NF449 (Gαs-selective antagonist). (l) Immunoblot analysis of phosphorylated CREB (p-CREB) and CREB in MIN6 cells treated with 100 μg/mL sm-apoA-IV alone, or 100 μg/mL sm-apoA-IV together with 20 μmol/L of SQ22536. HSP90 is a loading control. (m) Ratio of p-CREB/HSP90 calculated from l. Data are presented as mean ± SEM. ^*^P < 0.05, ^**^P < 0.01, ^***^P < 0.001. ns, no significance. GSIS, glucose-stimulated insulin secretion. STZ, streptozocin. sh-apoA-IV, signal peptide-removed human apoA-IV. sm-apoA-IV, signal peptide-removed mouse apoA-IV.

As described above, apoA-IV may participate in glycemic control in humans, and apoA-IV was proven to improve glucose tolerance in rodents. Since normal insulin secretion from pancreatic islets plays a crucial role in maintaining glucose homeostasis in the body, we wondered whether apoA-IV affected insulin secretion of pancreatic islets. Notably, apoA-IV significantly promoted GSIS in isolated non-diabetic human primary islets (Fig. 3a), indicating that apoA-IV functions in glycemic control in humans through affecting insulin secretion of pancreatic islets. Similarly, following apoA-IV treatment, GSIS was observed to be enhanced in both isolated primary islets from spontaneous T2D KKAy mice and mouse pancreatic β-cell line MIN6 (mouse insulinoma) (Fig. 3b and c). Additionally, administered recombinant apoA-IV-Flag (Flag tagged to C-terminal of apoA-IV) was detected in mouse pancreas 1 h after intraperitoneal administration but not detectable in mouse brain (arrowhead shown in Fig. 3d). Fluorescent sections of pancreas and brain tissue were observed to ascertain the distribution of apoA-IV in these tissues. In alignment with the immunoblot results, apoA-IV-GFP was predominantly localized in the pancreas and was scarcely detectable in the brain (Fig. 3e). These results demonstrated that one of the mechanisms by which apoA-IV exerts its glycemic control effects is by promoting insulin secretion in pancreatic β-cells. Indeed, in the case of type 1 diabetic (T1D) mice, where islet β cells were destroyed using streptozotocin (STZ) (Fig. 3f), the administration of apoA-IV did not lead to improved glucose tolerance (Fig. 3g).

Further, we explored the mechanism by which apoA-IV enhanced the secretion of insulin in pancreatic islets. It is known that cAMP and Ca^2+^ play an important role in regulating GSIS from islet β cells. Additionally, Gαq, Gαi, and Gαs-coupled GPCRs are known to mediate signaling pathways in islet β cells [22]. Therefore, [cAMP]_i_, [DAG]_i_, and [Ca^2+^]_i_ in MIN6 cells were examined after apoA-IV treatment. As shown in Fig. 3h, apoA-IV treatment significantly increased cytoplasmic cAMP content in MIN6 cells. However, it did not affect [DAG]_i_ and [Ca^2+^]_i_ in MIN6 cells (Fig. 3i and j). Both G protein Gαs and adenylate cyclase (AC) are intimately involved in the formation of cAMP. We chose AC inhibitor SQ22536 and Gαs-selective antagonist NF449 to test the effect of apoA-IV treatment on GSIS. Both SQ22536 and NF449 could partially block the stimulating effect evoked by apoA-IV in MIN6 cells (Fig. 3k). Additionally, apoA-IV treatment slightly promoted the phosphorylation of cAMP-response element binding protein (CREB) (Fig. 3l and m).

These results showed that apoA-IV acts on islet β cells to promote insulin secretion partially via the Gαs-coupled GPCR/cAMP pathway.

### ApoA-IV enhances energy expenditure

**Figure 4:**
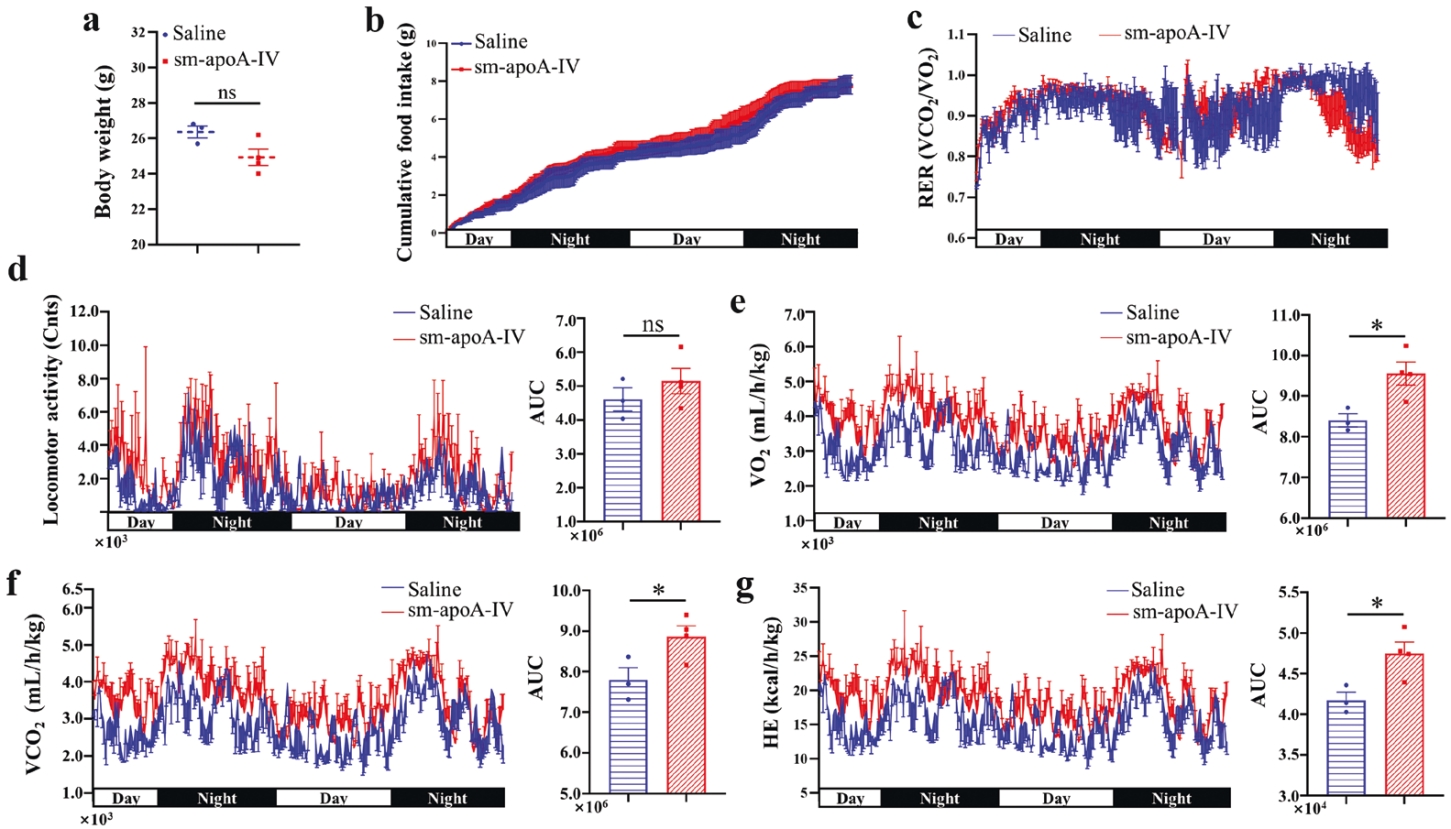
ApoA-IV enhances energy expenditure. Analysis of indirect calorimetry of WT male mice following the administration of saline (*n* = 3) or sm-apoA-IV (*n* = 4). (a) Body weight. (b) Cumulative food intake. (c) Respiratory exchange ratio (RER). (d) Locomotor activity. (e) O_2_ consumption (VO_2_). (f) CO_2_ production (VCO_2_). (g) Heat expenditure (HE). The right panel is the area under the curve (AUC) in the left panel (d−g). Data are presented as mean ± SEM. Statistical significance was determined by the two-tailed Student’s *t*-test. ^*^P < 0.05, two-tailed *t*-test. ns, no significance. sm-apoA-IV, signal peptide-removed mouse apoA-IV.

Since glucose metabolism plays a vital role in energy homeostasis in living organisms, we then investigated whether apoA-IV would affect energy homeostasis in the body. ApoA-IV-treated mice were subjected to indirect calorimetry. There was no difference in body weight between the two groups (Fig. 4a). Inconsistent with a previous report that apoA-IV inhibits appetite [23, 24], there was no discernible decrease in cumulative food intake after administration of eukaryote-derived apoA-IV (Fig. 4b). Although there were no comparable differences in the respiratory exchange ratio (RER) and locomotor activity between the two groups (Fig. 4c and d), apoA-IV-treated mice exhibited a dramatic increase in oxygen consumption (VO_2_), carbon dioxide production (VCO_2_), and heat expenditure (HE) (Fig. 4e−g). Consistent with this observation, the metabolic rate of apoA-IV-treated *db/db* mice was also elevated (Supplementary Fig. S3). Actually, it has been reported that diet-induced brown adipose tissue (BAT) thermogenesis and energy expenditure are reduced in apoA-IV knockout (apoA-IV-KO) mice [25].

Collectively, these results showed that apoA-IV enhances energy expenditure independent of food intake, which may be associated with improved glucose tolerance.

### T55−121 of murine apoA-IV improves glucose tolerance

**Figure 5:**
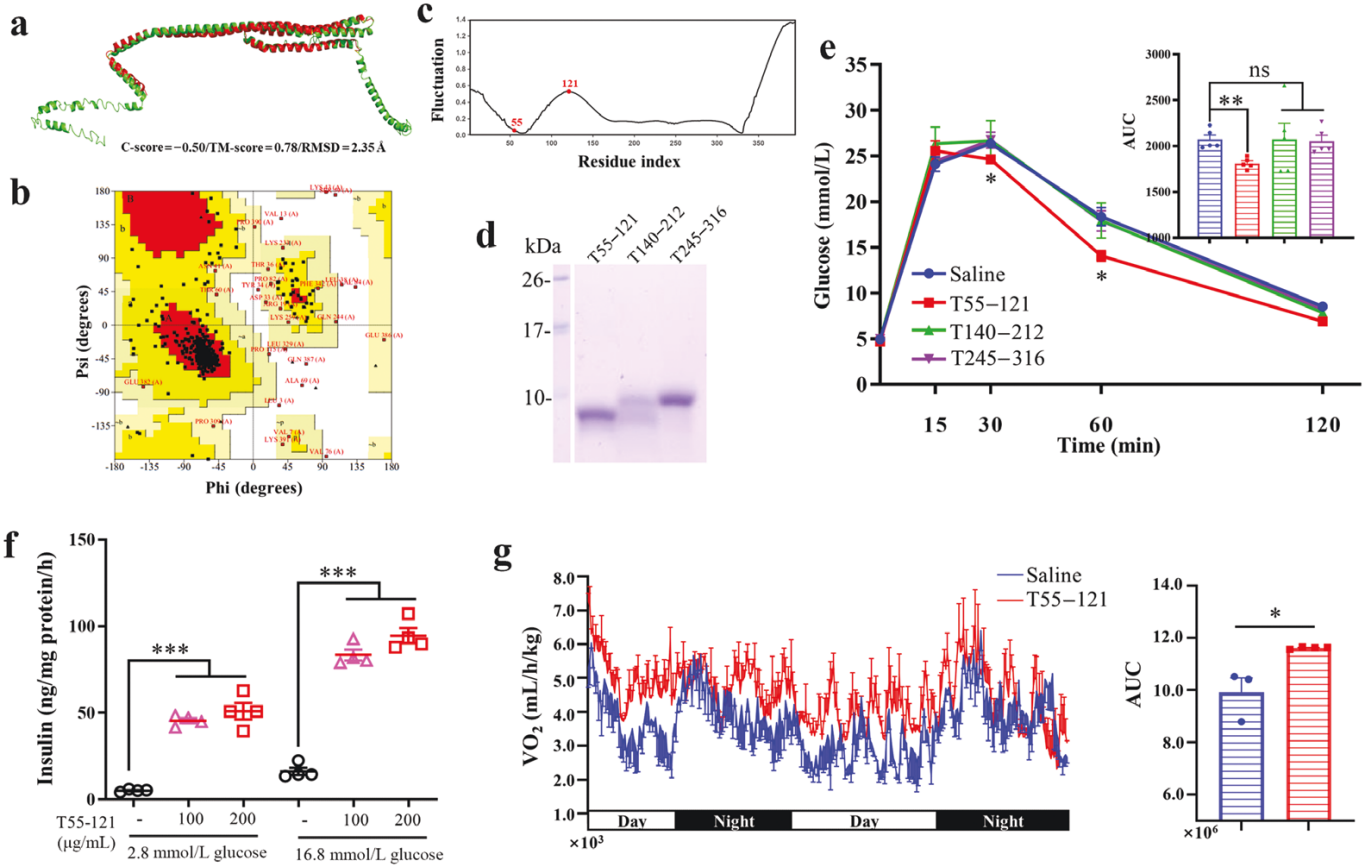
T55−121 derived from apoA-IV improves glucose tolerance. (a−c) Bioinformatics analysis to predict functional peptides of apoA-IV. Model 1 of the 3D structure of mouse apoA-IV (a). Ramachandran plot shows the distributions of phi and psi angles with z-score of apoA-IV Model 1 (b). Fluctuation profiles in the second slowest mode for apoA-IV (c). (d) SDS-PAGE of the peptides synthesized by solid-phase synthesis methods. (e) The IPGTT in WT mice (*n* = 5) with saline, T55−121, T140−212, or T245−316 (10 mg/kg body weight) treatment. (f) GSIS assay in MIN6 cells treated with 0, 100, or 200 μg/mL T55−121. (g) VO_2_ of WT mice following the administration of saline or T55−121. The right panel is the AUC in the left panel. Data are presented as mean ± SEM. ^*^P < 0.05, ^**^P < 0.01, ^***^P < 0.001.

Identifying the specific functional peptide of apoA-IV that is involved in modulating glucose homeostasis can have significant therapeutic implications for managing obesity and T2D. We employed the Gaussian network model (GNM) to predict the potential functional peptide of apoA-IV. The iterative threading assembly refinement (I-TASSER) server was used to predict the mouse apoA-IV model and Model 1 in Fig. 5a exhibited superior characteristics compared to the other models, as indicated by its C-score and Ramachandran plot (Fig. 5b). Notably, Model 1 demonstrated satisfactory stereochemical quality, with only 3% of residues falling within the disallowed region of the Ramachandran plot (Fig. 5b). Besides, numerous studies have documented the utilization of the slowest motion mode of GNM to identify crucial functional residues in catalytic sites [26] and ligand-binding sites [27]. The fluctuation profiles of mouse apoA-IV were exhibited in the second slowest mode. The significant fluctuations were considered to play a driving role in protein conformation transitions. As depicted in Fig. 5c, the residue 55 resided near the minima and the residue 121 was located at the peak of the second slowest mode. The calculation results indicated that T55−121 may be a potential functional peptide of mouse apoA-IV.

Thus, peptide T55−121 and other two peptides (T140−212 and T245−316) of mouse apoA-IV were synthesized using solid-phase synthesis method (Fig. 5d). The glucose tolerance test showed that only the treatment with T55−121 resulted in improved glucose tolerance while the other two peptides did not exhibit any effects (Fig. 5e). Furthermore, T55−121 was revealed to promote insulin secretion in MIN6 cells (Fig. 5f). In line with the effect of full-length apoA-IV on enhancing energy expenditure, T55−121 significantly increased oxygen consumption (Fig. 5g).

These results showed that the murine T55−121 of apoA-IV exhibits the ability to improve glucose tolerance and increase oxygen consumption.

## Discussion

Bariatric/metabolic surgery is currently the most effective strategy for treating patients with severe obesity and T2D. The procedure results in metabolic benefits through multiple mechanisms including caloric restriction, elevated glucagon-like peptide-1 (GLP-1) levels, altered bile acids, and an altered microbiome composition [28, 29]. Both RYGB and LSG have similar benefits of durable weight loss, reduced hyperlipidemia, and improved glucose homeostasis [30]. Therefore, it is crucial to identify the key factors that undergo significant changes following bariatric surgeries to enhance our understanding of the mechanisms involved in glucose control. In this study, through gel-based proteomics and immunoblotting analysis, a dramatic increase in serum apoA-IV levels in patients following LSG is demonstrated, consistent with results in other bariatric surgeries [11, 12, 19, 31]. Therefore, apoA-IV may play an important role in glycemic control in humans. Indeed, for the first time, we have demonstrated that apoA-IV could enhance insulin secretion in human islets (Fig. 3).

In the current study, we observed that both the administration of recombinant apoA-IV protein (Fig. 2; Supplementary Fig. S4) and the *in vivo* overexpression of apoA-IV using AAV (Fig. 3) led to significant improvements in glucose tolerance in various diabetic rodent models. However, there appear to be contradictory phenotypes in apoA-IV-deficient mice and rats. ApoA-IV-KO mice displayed impaired glucose tolerance [15], whereas apoA-IV-deficient rats exhibited the opposite phenotype with improved glucose clearance [32]. The discrepancy could potentially be attributed to variations in host species and the specific gene knockout procedures. Further investigation is needed to clarify these contrasting outcomes.

Regarding the potential mechanism of apoA-IV in regulating glucose homeostasis, we demonstrated that apoA-IV promoted GSIS in islet cells, partially via a Gαs-coupled GPCR/cAMP pathway. Regarding the apoA-IV receptor, Li *et al.* found that apoA-IV directly interacts with nuclear receptors NR1D1 and NR4A1, to reduce gluconeogenesis in hepatic cells [16, 17]. In another work, it was found that apoA-IV can bind to apoA-I or apoA-II recognition sites at the cell surface to regulate cholesterol efflux in adipose cells [33]. It was recently reported that apoA-IV binds LDL receptor-related protein 1 (LRP1) to facilitate glucose uptake in adipose tissue [34]. In summary, there may be multiple binding sites for apoA-IV, contributing to the regulation of metabolism.

Several mimetic peptides derived from apolipoproteins have been investigated and developed as potential therapeutic agents [35, 36]. ApoA-I mimetic peptides promote the formation of HDLs and facilitate cholesterol efflux [37, 38]. ApoE mimetic peptides bind to LRPs and play a significant role in reducing inflammation and preventing cardiovascular diseases as well as neurodegenerative diseases [39–41]. Here, we discovered that T55−121 was a functional peptide of murine apoA-IV, which can improve glucose tolerance, providing a foundation for developing possible therapeutic interventions targeting glucose-related disorders. Furthermore, denatured apoA-IV showed a similar effect to the native form, leading to improved glucose tolerance. This suggests that apoA-IV and the identified T55−121 peptide may perform their biological functions independently of their secondary structure (Supplementary Fig. S5). Further, we performed an alignment analysis of the apoA-IV functional peptide T55−121 with other apolipoprotein mimetic peptides, including apoA-I, apoA-E, and apoC-II. The alignment result did not reveal any common features among these peptides (Supplementary Fig. S6). Understanding the differences and unique characteristics of those mimetic peptides will contribute to a more comprehensive understanding of their functions and therapeutic potentials.

Bariatric/metabolic surgeries benefiting patients with severe T2D offer insight into factors involved in maintaining glucose homeostasis. In this study, serum proteomic analysis revealed a distinct protein profile in patients after LSG, with a significant increase in apoA-IV levels, and similar effects were observed in patients after RYGB. ApoA-IV may play a critical role in glycemic control, potentially by enhancing insulin secretion in human islets. ApoA-IV and its derived peptide, T55−121, were demonstrated to improve energy expenditure and glucose tolerance in diabetic rodents, partially through Gαs-coupled GPCR/cAMP signaling. These findings enhance our understanding of apoA-IV’s connection to glycemic control, emphasizing its potential as a biomarker or therapeutic target for improving glucose regulation.

## Materials and methods

### Materials

The commercial materials and reagents used in the present study are listed in Supplementary Table S4 and were applied as the manufacturers’ procedures described. The peptides of apoA-IV were synthesized by GL Biochem (Shanghai) Ltd.

### Human specimen

The blood samples were collected from two diabetic obese patients before and one year after LSG. Patient characteristics are listed in Supplementary Table S5. The use of human blood samples was approved by the Ethics Committee of the First Affiliated Hospital, College of Medicine, Zhejiang University. Human islets from nondiabetic donors were isolated through Collagenase NB1 and Neutral Protease NB digestion followed by continuous density purification [42]. Then islets were picked up by hand and cultured in CMRL-1066 medium, supplemented with 10% human serum albumin at 37°C in 5% CO_2_. The characteristics of nine non-diabetic subjects are shown in Supplementary Table S6. The use of human islets was approved by the Ethics Committee of the Tianjin First Central Hospital. All procedures were performed in accordance with institutional guidelines and written informed consents were obtained from all the participants.

### Liquid chromatography with tandem mass spectrometry (LC–MS/MS) analysis for protein identification

The sera collected from patients were precipitated by the addition of a 1.5-fold volume of acetonitrile followed by vortex mixing. The samples were centrifuged at 20,000 *g* for 5 min at 4ºC and the supernatants were collected for evaporation using a CentriVap Benchtop Vacuum Concentrator (LABCONCO, USA). The samples were separated by SDS–PAGE and were stained with Coomassie Brilliant Blue according to the manufacturers’ procedure. The protein bands of interest were cut and subjected to enzymatic hydrolysis. The peptide mixture was analyzed by the Laboratory of Proteomics, Institute of Biophysics, Chinese Academy of Sciences.

### Animals

WT C57BL/6J mice were obtained from Vital River Laboratories (Beijing, China). Diabetic Lepr^db^ (*db/db*) mice and obese Lep^ob^ (*ob/ob*) mice were purchased from Nanjing Biomedical Research Institute of Nanjing University. Spontaneous T2D KKAy mice were obtained from Beijing HFK Bioscience Co. Ltd (Beijing, China), and GK diabetic rats were generously supplied by Prof. Tao Xu’s Lab. All animals were maintained under a 12-h light/12-h dark cycle at 22 ± 1°C. The KKAy mice were fed with a high-fat diet (Research Diets, Inc., D12451, USA), and the others were fed with standard rodent chow. All mice were used between 6 and 24 weeks of age. All animal experiment procedures were approved by the Animal Care and Use Committee of the Institute of Biophysics, Chinese Academy of Sciences.

### Construction of animal models

For apoA-IV-overexpressing mice, 16-week-old *ob/ob* mice were infected with AAV encoding mouse apoA-IV (apoA-IV-AAV) or control AAV encoding GFP (Con-AAV) via intraperitoneal injection. To mimic the expression of endogenous apoA-IV, an AAV9 expressing apoA-IV-Flag under a CMV promoter was constructed, which was administered via intraperitoneal injection to infect the small intestine. The mice received a single dose of 2.0 × 10^12^ vector genome (vg) doses of AAV9. ApoA-IV-AAV and Con-AAV were produced by Hanbio Biotechnology Co., LTD (Shanghai, China). The T1D mouse model induced by STZ was obtained as described previously [43]. STZ was dissolved to 0.1 mol/L in sodium citrate buffer (pH = 4.2). Ten-week-old WT C57BL/6J mice were injected once intraperitoneally with STZ (150 mg/kg body weight) and were used when their random blood glucose level was constantly above 20 mmol/L.

### Intraperitoneal glucose tolerance test (IPGTT)

For the IPGTT, mice were fasted for 16 h and injected intraperitoneally with saline or apoA-IV. Two hours later, glucose was injected intraperitoneally (2 g/kg body weight). Tail blood samples were collected and glucose levels were measured with a commercial glucometer (Ascensia ELITE; Bayer) at 0, 15, 30, 60, and 120 min after challenge.

### Measurement of indirect calorimetry

WT mice or *db/db* mice, fasted overnight for 16 h, were administered saline or apoA-IV intraperitoneally. Two hours after injection, indirect calorimetry was performed using a TSE Systems instrument (Germany) following the manufacturer’s procedure. The data were analyzed using GraphPad Prism.

### Primary islet preparation

The islets of KKAy mice were isolated via the collagenase V perfusion method and were cultured in RPMI-1640 medium supplemented with 10% fetal bovine serum (FBS). The detailed procedure was reported previously [44].

### Cell culture

The mouse β cell line MIN6 was a gift of Prof. Xiao Han at Nanjing Medical University. The cells were cultured in Dulbecco’s modified Eagle medium (DMEM) supplemented with 15% FBS.

### Expression and purification of recombinant apoA-IV protein

The construction of the prokaryotic expression system was based on the method described in a previous report [23]. The eukaryotic expression system was constructed to obtain endotoxin-free recombinant apoA-IV. The nucleotide sequence of human serum albumin signal peptide (SP_HSA_) was inserted at the 5ʹ end of HIS-SMT3-apoA-IV to improve yield. HEK293F suspension cells were transiently transfected with the pcDNA3.1-SP_HSA_-HIS-SMT3-apoA-IV plasmid. The secreted apoA-IV was purified with Ni Sepharose 6 Fast Flow chromatography according to the manufacturer’s guidelines. The recombinant apoA-IV from the eukaryotic expression system constructed by our laboratory was only used in glucose tolerance tests in WT mice. Endotoxin-free recombinant apoA-IV used in other experiments was obtained from DetaiBio Co., Ltd (Nanjing, China).

### Immunoblot analysis

For the immunoblot analysis, proteins were dissolved in 2 × sample buffer, denatured at 95°C for 5 min, separated by SDS-PAGE, and transferred to a PVDF membrane. The membrane was incubated in primary and then secondary antibodies (listed in Supplementary Table S4). The image was detected by an ECL chemiluminescence system (SAGECREATION, China). The intensities of bands were analyzed by the software Image J.

### Fluorescence section of tissues

The pancreas and brain of GK diabetic rats (4 weeks) were dissected and frozen in OCT Tissue Tek Compound, and cut into 5 μm and 15 μm thick sections, respectively. They were incubated with 10 μg/mL apoA-IV-GFP or GFP at room temperature for 2 h and then washed three times with phosphate-buffered saline (PBS). The signals were detected by an inverted fluorescence microscope (Zeiss).

### Insulin measurement

MIN6 cells, human primary islets, and mouse primary islets were used to test insulin secretion in response to different concentrations of glucose [44]. MIN6 cells were treated with vehicle, 100 μg/mL sm-apoA-IV, or sm-apoA-IV together with SQ22536 or NF449 for 3 h. Isolated human primary islets and mouse primary islets were treated with vehicle, 100 μg/mL sh-apoA-IV, or sm-apoA-IV for 3 h. The GSIS assay was carried out in Kreb’s buffer containing 2.8 or 16.8 mmol/L glucose. The supernatant was collected for measurement of insulin by enzyme-linked immunosorbent assay (ELISA), and cells and islets were used for quantitative determination of protein by BCA protein assay kit.

### Measurement of [cAMP]_i_, [DAG]_i_, and [Ca^2+^]_i_

For measurement of [cAMP]i and [DAG]i, MIN6 cells were transfected with a cAMP or diacylglycerol (DAG) green downward fluorescent sensor and cultured for 24 h [45]. Then the cells were treated with 0, 100, or 200 μg/mL sm-apoA-IV for 3 h. The fluorescence of the cAMP sensor or DAG sensor was detected after treatment with 2.8 mmol/L or 16.8 mmol/L glucose using a PerkinElmer EnSpire2300 Multimode Microplate Reader (Synergy 2, BIO-TEK, USA). For measurement of [Ca^2+^]_i_, MIN6 cells in 96-well plates were cultured with 0, 100, or 200 μg/mL sm-apoA-IV for 1 h. Then they were loaded with the Ca^2+^ fluorescent sensor Fluo-4 AM for 30 min at 37°C. The cells in Krebs buffer containing 2.8 mmol/L glucose were the basic situation. For stimulation, the cells were perfused with Krebs buffer containing 16.8 mmol/L glucose with vehicle or sm-apoA-IV. The changes in [Ca^2+^]_i_ were recorded using a Multimode Microplate Reader through a time-series recording of the fluorescent signal [46].

### The prediction of the potential functional peptide of apoA-IV

The modeling approaches and the GNM were used to predict the potential functional peptide of apoA-IV [47]. The Protein Data Bank (PDB) was used for searching the structure of mouse apoA-IV [48]. Since the experimental 3D structure of mouse apoA-IV is unavailable, a 3D model of mouse apoA-IV was constructed using I-TASSER [49], with the structure of human apoA-IV (PDB ID: 3S84) as a template [50]. The selection of the model was based on two criteria, including the C-score indicated by the confidence score provided by the I-TASSER server and stereochemical properties evaluated using the PROCHECK tool. In the GNM, the 3D structure of a protein is considered as an elastic network of Cα atoms, in which nodes are connected by harmonic springs with a force constant γ. The internal energy of the system in the GNM is defined as


$$\begin{array}{l} V=\frac{1}{2}\gamma \left[\Delta R^{T}(\Gamma ⊗E)\Delta R\right] \end{array}$$

where ΔR represents the 3*N*-dimensional column vector of fluctuation $\Delta R_{1}$, $\Delta R_{2}$, … $\Delta R_{N}$ of the C_*α*_ atoms, where *N* is the number of residues; the superscript T denotes the transpose; ***E*** is the third-order identity matrix; ⊗ is the direct product; and **Г** is the *N* × *N* symmetric Kirchhoff matrix.

In the GNM, the topology of the network is defined by the *N × N* Kirchhoff matrix Г, the elements of which are described as


$$\begin{array}{l} \Gamma_{ij}=\left\{ \begin{array}{llll} & \;-1,\;if\;i\neq j\;and\;R_{ij}\;\;\leq r_{c}\; & \;0,\;if\;i\neq j\;and\;R_{ij}>r_{c}\; & -\sum_{j,\;j\neq i}^{N}\Gamma_{ij},\;if\;i=j\; \end{array}\right.\; \end{array}$$

where *r*_*c*_ is the cutoff distance and *R*_*ij*_ is the distance between the *i*th and *j*th nodes.

The mean-square fluctuation of each residue is in proportion to the diagonal elements of the pseudoinverse of the Kirchhoff matrix.


$$\begin{array}{l} 〈\Delta R_{i}\cdot \Delta R_{i}〉=\frac{3k_{B}T}{\gamma }{\left[\Gamma^{-1}\right]}_{ii} \end{array}$$

where *k*_*B*_ is the Boltzmann constant and *T* is the thermodynamic temperature. The pseudoinverse of the Kirchhoff matrix can be decomposed as


$$\begin{array}{l} \Gamma^{-1}\;=\;\sum_{k=2}^{N}\lambda_{k}^{-1}\mu_{k}\cdot \mu {}_{k}^{T} \end{array}$$

where *λ*_*k*_ and ***μ***_*k*_ are the *k*th eigenvalue and eigenvector of the Kirchhoff matrix, respectively.

### Statistical analysis

Results are presented as mean ± SEM. Statistical significance was determined by the two-tailed Student’s *t*-test between two groups or ANOVA in multiple groups. *P* < 0.05 was considered to be a significant difference.

## Supplementary Material

loae010_suppl_Supplementary_Materials

## Data Availability

All study data are included in the article and/or Supplementary Materials. Materials are available upon request.
