# Supplementary material for: Apolipoprotein A-IV and its derived peptide, T55−121, improve glycemic control and increase energy expenditure
Source: Life Metab. 2024 Mar 14;3(4):loae010. doi: 10.1093/lifemeta/loae010 (PMC11748984; doi:10.1093/lifemeta/loae010)
Supplement: loae010_suppl_Supplementary_Materials [file loae010_suppl_Supplementary_Materials.pdf]

Supplementary Table S1 MS results of the band 1 from patient after LSG.

Number	Accession	Description	Score	MW [kDa]	Coverage	Proteins	Unique Peptides	Peptides	PSMs	AAs	calc. pI
1	P06727	Apolipoprotein A-IV OS=Homo sapiens GN=APOA4 PE=1 SV=3 - [APOA4_HUMAN]	184.90	45.4	61.36	2	28	28	110	396	5.38
2	P02763	Alpha-1-acid glycoprotein 1 OS=Homo sapiens GN=ORM1 PE=1 SV=1 - [A1AG1_HUMAN]	118.55	23.5	48.26	1	8	11	112	201	5.02
3	P19652	Alpha-1-acid glycoprotein 2 OS=Homo sapiens GN=ORM2 PE=1 SV=2 - [A1AG2_HUMAN]	63.15	23.6	35.82	1	5	8	61	201	5.11
4	P0C0L4-2	Isoform 2 of Complement C4-A OS=Homo sapiens GN=C4A - [CO4A_HUMAN]	38.25	187.6	6.83	4	3	11	26	1698	7.12
5	F5GXS0	C4b-B OS=Homo sapiens GN=C4B PE=4 SV=1 - [F5GXS0_HUMAN]	37.21	187.6	6.83	2	3	11	27	1698	7.33
6	P25311	Zinc-alpha-2-glycoprotein OS=Homo sapiens GN=AZGP1 PE=1 SV=2 - [ZA2G_HUMAN]	30.22	34.2	36.24	3	12	12	19	298	6.05
7	P60709	Actin, cytoplasmic 1 OS=Homo sapiens GN=ACTB PE=1 SV=1 - [ACTB_HUMAN]	29.93	41.7	33.60	21	4	10	34	375	5.48
8	P02765	Alpha-2-HS-glycoprotein OS=Homo sapiens GN=AHSG PE=1 SV=1 - [FETUA_HUMAN]	21.47	39.3	17.44	3	5	5	9	367	5.72
9	P01009-2	Isoform 2 of Alpha-1-antitrypsin OS=Homo sapiens GN=SERPINA1 - [A1AT_HUMAN]	14.94	40.2	24.79	7	7	7	13	359	5.47
10	P02766	Transthyretin OS=Homo sapiens GN=TTR PE=1 SV=1 - [TTHY_HUMAN]	11.51	15.9	18.37	1	3	3	5	147	5.76
11	F5H0C8	Enolase OS=Homo sapiens GN=ENO2 PE=2 SV=1 - [F5H0C8_HUMAN]	8.01	34.7	6.98	8	1	1	3	315	4.87
12	P02750	Leucine-rich alpha-2-glycoprotein OS=Homo sapiens GN=LRG1 PE=1 SV=2 - [A2GL_HUMAN]	7.93	38.2	11.24	1	4	4	5	347	6.95
13	P05090	Apolipoprotein D OS=Homo sapiens GN=APOD PE=1 SV=1 - [APOD_HUMAN]	7.26	21.3	13.23	4	2	2	4	189	5.15
14	P68032	Actin, alpha cardiac muscle 1 OS=Homo sapiens GN=ACTC1 PE=1 SV=1 - [ACTC_HUMAN]	5.74	42.0	26.79	16	2	8	17	377	5.39
15	P02647	Apolipoprotein A-I OS=Homo sapiens GN=APOA1 PE=1 SV=1 - [APOA1_HUMAN]	4.87	30.8	8.99	2	2	2	3	267	5.76
16	E5RK62	SPARC (Fragment) OS=Homo sapiens GN=SPARC PE=2 SV=1 - [E5RK62_HUMAN]	4.18	13.4	16.52	3	2	2	3	115	7.25
17	P81605	Dermcidin OS=Homo sapiens GN=DCD PE=1 SV=2 - [DCD_HUMAN]	3.54	11.3	10.00	2	1	1	2	110	6.54
18	P12259	Coagulation factor V OS=Homo sapiens GN=F5 PE=1 SV=4 - [FA5_HUMAN]	2.33	251.5	0.49	1	1	1	1	2224	6.05
19	P01019	Angiotensinogen OS=Homo sapiens GN=AGT PE=1 SV=1 - [ANGT_HUMAN]	2.29	53.1	7.63	1	3	3	3	485	6.32
20	P02652	Apolipoprotein A-II OS=Homo sapiens GN=APOA2 PE=1 SV=1 - [APOA2_HUMAN]	2.04	11.2	21.00	1	3	3	3	100	6.62
21	Q5VY30	Plasma retinol-binding protein(1-182) OS=Homo sapiens GN=RBP4 PE=2 SV=1 - [Q5VY30_HUMAN]	1.60	22.9	5.03	2	1	1	1	199	6.09
22	H7C1V2	RalBP1-associated Eps domain-containing protein 1 (Fragment) OS=Homo sapiens GN=REPS1 PE=2 SV=1 - [H7C1V2_HUMAN]	0.00	16.5	23.13	1	1	1	1	147	5.02
23	O75643	U5 small nuclear ribonucleoprotein 200 kDa helicase OS=Homo sapiens GN=SNRNP200 PE=1 SV=2 - [U520_HUMAN]	0.00	244.4	0.47	1	1	1	1	2136	6.06
24	O95445-2	Isoform 2 of Apolipoprotein M OS=Homo sapiens GN=APOM - [APOM_HUMAN]	0.00	13.0	16.38	3	2	2	2	116	7.75
25	Q562R1	Beta-actin-like protein 2 OS=Homo sapiens GN=ACTBL2 PE=1 SV=2 - [ACTBL_HUMAN]	0.00	42.0	12.77	1	1	4	6	376	5.59
26	Q6UXD5-6	Isoform 6 of Seizure 6-like protein 2 OS=Homo sapiens GN=SEZ6L2 - [SE6L2_HUMAN]	0.00	86.8	1.24	5	1	1	1	809	4.82

Supplementary Table S2 MS results of the band 2 from patient after LSG.

Number	Accession	Description	Score	MW [kDa]	Coverage	Proteins	Unique Peptides	Peptides	PSMs	AAs	calc. pI
1	P06727	Apolipoprotein A-IV OS=Homo sapiens GN=APOA4 PE=1 SV=3 - [APOA4_HUMAN]	123.85	45.4	57.32	2	25	25	84	396	5.38
2	P02763	Alpha-1-acid glycoprotein 1 OS=Homo sapiens GN=ORM1 PE=1 SV=1 - [A1AG1_HUMAN]	120.72	23.5	45.27	1	6	9	96	201	5.02
3	P19652	Alpha-1-acid glycoprotein 2 OS=Homo sapiens GN=ORM2 PE=1 SV=2 - [A1AG2_HUMAN]	55.85	23.6	35.82	1	5	8	46	201	5.11
4	P0C0L4-2	Isoform 2 of Complement C4-A OS=Homo sapiens GN=C4A - [CO4A_HUMAN]	34.32	187.6	7.89	4	2	11	26	1698	7.12
5	F5GXS0	C4b-B OS=Homo sapiens GN=C4B PE=4 SV=1 - [F5GXS0_HUMAN]	31.87	187.6	7.89	2	2	11	26	1698	7.33
6	P25311	Zinc-alpha-2-glycoprotein OS=Homo sapiens GN=AZGP1 PE=1 SV=2 - [ZA2G_HUMAN]	24.21	34.2	33.22	2	10	10	17	298	6.05
7	P60709	Actin, cytoplasmic 1 OS=Homo sapiens GN=ACTB PE=1 SV=1 - [ACTB_HUMAN]	20.27	41.7	32.80	23	4	9	16	375	5.48
8	P02765	Alpha-2-HS-glycoprotein OS=Homo sapiens GN=AHSG PE=1 SV=1 - [FETUA_HUMAN]	14.28	39.3	9.26	2	2	2	6	367	5.72
9	Q5T8M8	Actin, alpha skeletal muscle OS=Homo sapiens GN=ACTA1 PE=2 SV=1 - [Q5T8M8_HUMAN]	8.30	32.0	26.13	16	1	6	8	287	5.41
10	P01009-2	Isoform 2 of Alpha-1-antitrypsin OS=Homo sapiens GN=SERPINA1 - [A1AT_HUMAN]	8.10	40.2	21.73	4	5	5	7	359	5.47
11	O95445-2	Isoform 2 of Apolipoprotein M OS=Homo sapiens GN=APOM - [APOM_HUMAN]	6.20	13.0	11.21	3	1	1	3	116	7.75
12	P05090	Apolipoprotein D OS=Homo sapiens GN=APOD PE=1 SV=1 - [APOD_HUMAN]	5.68	21.3	17.46	3	3	3	3	189	5.15
13	C9JKR2	Albumin, isoform CRA_k OS=Homo sapiens GN=ALB PE=4 SV=1 - [C9JKR2_HUMAN]	5.61	47.3	6.00	7	1	3	5	417	6.35
14	P15085	Carboxypeptidase A1 OS=Homo sapiens GN=CPA1 PE=1 SV=2 - [CBPA1_HUMAN]	4.80	47.1	5.49	3	2	2	2	419	5.76
15	P02750	Leucine-rich alpha-2-glycoprotein OS=Homo sapiens GN=LRG1 PE=1 SV=2 - [A2GL_HUMAN]	4.59	38.2	11.24	1	3	3	4	347	6.95
16	H3BUX1	Mesothelin (Fragment) OS=Homo sapiens GN=MSLN PE=2 SV=1 - [H3BUX1_HUMAN]	2.27	43.8	3.52	6	1	1	2	398	6.37
17	P02652	Apolipoprotein A-II OS=Homo sapiens GN=APOA2 PE=1 SV=1 - [APOA2_HUMAN]	2.24	11.2	11.00	1	1	1	1	100	6.62
18	P12259	Coagulation factor V OS=Homo sapiens GN=F5 PE=1 SV=4 - [FA5_HUMAN]	2.15	251.5	0.49	1	1	1	1	2224	6.05
19	P02647	Apolipoprotein A-I OS=Homo sapiens GN=APOA1 PE=1 SV=1 - [APOA1_HUMAN]	1.96	30.8	4.87	1	1	1	1	267	5.76
20	F5GXS5	Apolipoprotein F OS=Homo sapiens GN=APOF PE=2 SV=1 - [F5GXS5_HUMAN]	1.61	33.4	4.55	2	1	1	1	308	5.45
21	H7C5W5	Peripherin (Fragment) OS=Homo sapiens GN=PRPH PE=3 SV=1 - [H7C5W5_HUMAN]	0.00	22.9	3.00	21	1	1	1	200	5.29
22	P01019	Angiotensinogen OS=Homo sapiens GN=AGT PE=1 SV=1 - [ANGT_HUMAN]	0.00	53.1	2.47	1	1	1	2	485	6.32
23	Q5VY30	Plasma retinol-binding protein(1-182) OS=Homo sapiens GN=RBP4 PE=2 SV=1 - [Q5VY30_HUMAN]	0.00	22.9	5.03	2	1	1	1	199	6.09

Supplementary Table S3 The change of plasma apoA-IV levels in the patients before and one year after surgery.

Subject number	Before surgery	One year after surgery
1	1.142	2.046
2	1.398	1.948
3	0.974	1.497
4	0.906	1.674
5	0.995	1.486
6	0.914	1.180
7	0.922	1.812
8	1.085	1.414
9	1.063	1.810
10	0.898	1.607

Supplementary Table S4 Antibodies and reagents.

**Antibody**	**Product number**	**Manufacturer**
Anti-mouse apoA-IV	PAB967Mu01	CLOUD-CLONE
Anti-human apoA-IV	1D6B6	Cell Signaling Technology
Transferrin	A1448	ABclonal
Flag	F1804	Sigma-Aldrich
CREB	9192	Cell Signaling Technology
pCREB (Ser133)	9191	Cell Signaling Technology
GAPDH	AB2302	Merck Millipore
HRP-labeled goat anti-mouse IgG	ZB-2305	ZSGB-BIO
HRP-labeled goat anti-rabbit IgG	ZB-2301	ZSGB-BIO
Alexa Fluor 594-conjugated goat anti-rabbit IgG	ZF-0516	ZSGB-BIO
**Reagent or kit name**	**Product number**	**Manufacturer**
Dulbecco's Modified Eagle's Medium (DMEM)	CM10017	MACGENE
CMRL 1066 medium	21530-027	Invitrogen Life Technologies
RPMI-1640 medium	C22400500BT	Invitrogen Life Technologies
Fetal bovine serum (FBS)	10437-028	GIBCO
Penicillin-Streptomycin	CC004	MACGENE
Collagenase NB1	DS17455.03	SERVA
Neutral Protease NB	DS30303.01	SERVA
Collagenase V	C9263	Sigma-Aldrich
Bovine serum albumin	A1933	Sigma-Aldrich
Streptozotocin	S0130	Sigma-Aldrich
Polybrene	S2267	Sigma-Aldrich
Triton X-100	T9284	Sigma-Aldrich
Insulin	P3375	Beyotime
Isopropyl β-D-1-thiogalactopyranoside (IPTG)	0487	VWR AMRESCO
Ni Sepharose 6 Fast Flow	17526801	GE Healthcare
PVDF membrane	ISEQ00010	Merck Millipore
Coomassie brilliant blue	PA101	TIANGEN
Adenylate cyclase inhibitor SQ22536	HY-100396	MedChemExpress
Gsα-selective antagonist NF449	1391	ROCRIS Bioscience
Green Down cADDis cAMP Assay Kit	0200G	Montana Molecular
Rat/Mouse Insulin ELISA Kit	EZRMI-13K	Merck Millipore
Human Insulin ELISA Kit	27365	Mercodia
Pierce™ BCA Protein Assay Kit	23227	Thermo Fisher Scientific
Ca^{2+} sensing fluorescent probe Fluo-4 AM	F14201	Thermo Fisher Scientific

Supplementary Table S5 Information of subjects for LSG.

	Subject 1	Subject 2
Gender	Male	Male
Age (years)	25	23
Height (cm)	176	175
Weight (kg)	135	115
BMI (kg/m^2)	42.93	37.55

Supplementary Table S6 Characteristic of subjects for donating primary islets.

Age (years)	27-62
Body weight (kg)	78.3±18.2
BMI (kg/m^2)	26.1±5.2
C-peptide (ng/mL)	13.2±6.7
HbA1c (%)	5.3±0.3

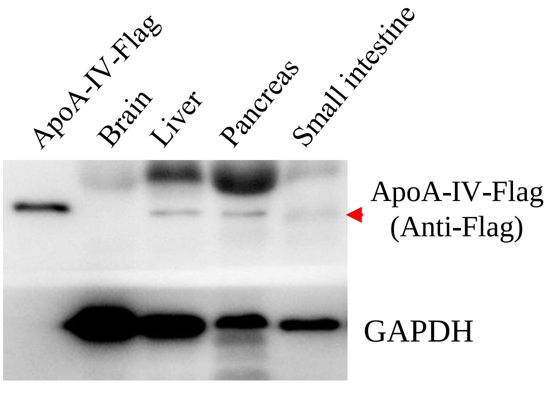

Supplementary Figure S1 The distribution of apoA-IV-Flag after AAV infection. Immunoblot analysis of apoA-IV-Flag in the brain, liver, pancreas, and small intestine in mouse infected with adeno-associated virus 9 expressing apoA-IV from a CMV promoter. Red arrowhead indicates apoA-IV-Flag.

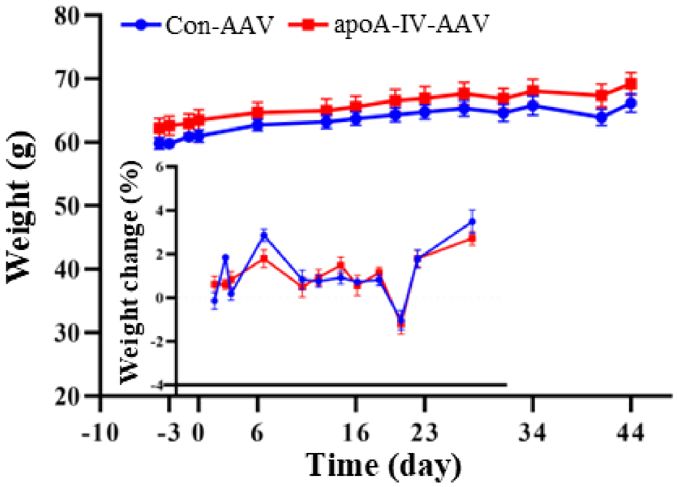

Supplementary Figure S2 Body weight in apoA-IV overexpressing ob/ob mice. Body weight and weight change (inlay on the lower left) of the Con-AAV infection group ($n = 6$) and apoA-IV-AAV infection group ($n = 6$).

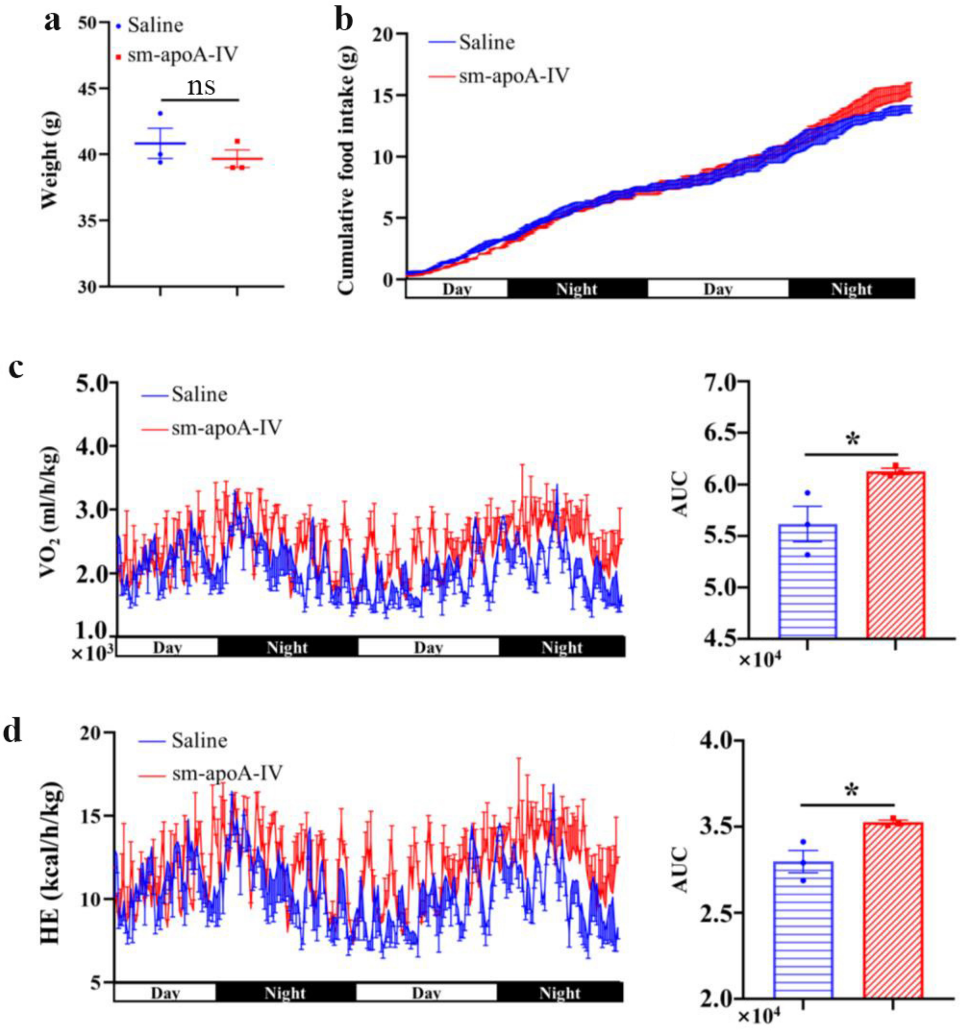

Supplementary Figure S3 ApoA-IV enhances energy expenditure in *db/db* mice. Analysis of indirect calorimetry of db/db mice following the administration of saline or sm-apoA-IV ($n = 3$). (a) Body weight. (b) Cumulative food intake. (c) O_2 consumption (VO_2). (d) Heat expenditure (HE). The right panel is the area under curve (AUC) (c and d). Data are presented as mean ± SEM. Statistical significance was determined by the two-tailed Student's *t*-test. $^{*}P < 0.05$.

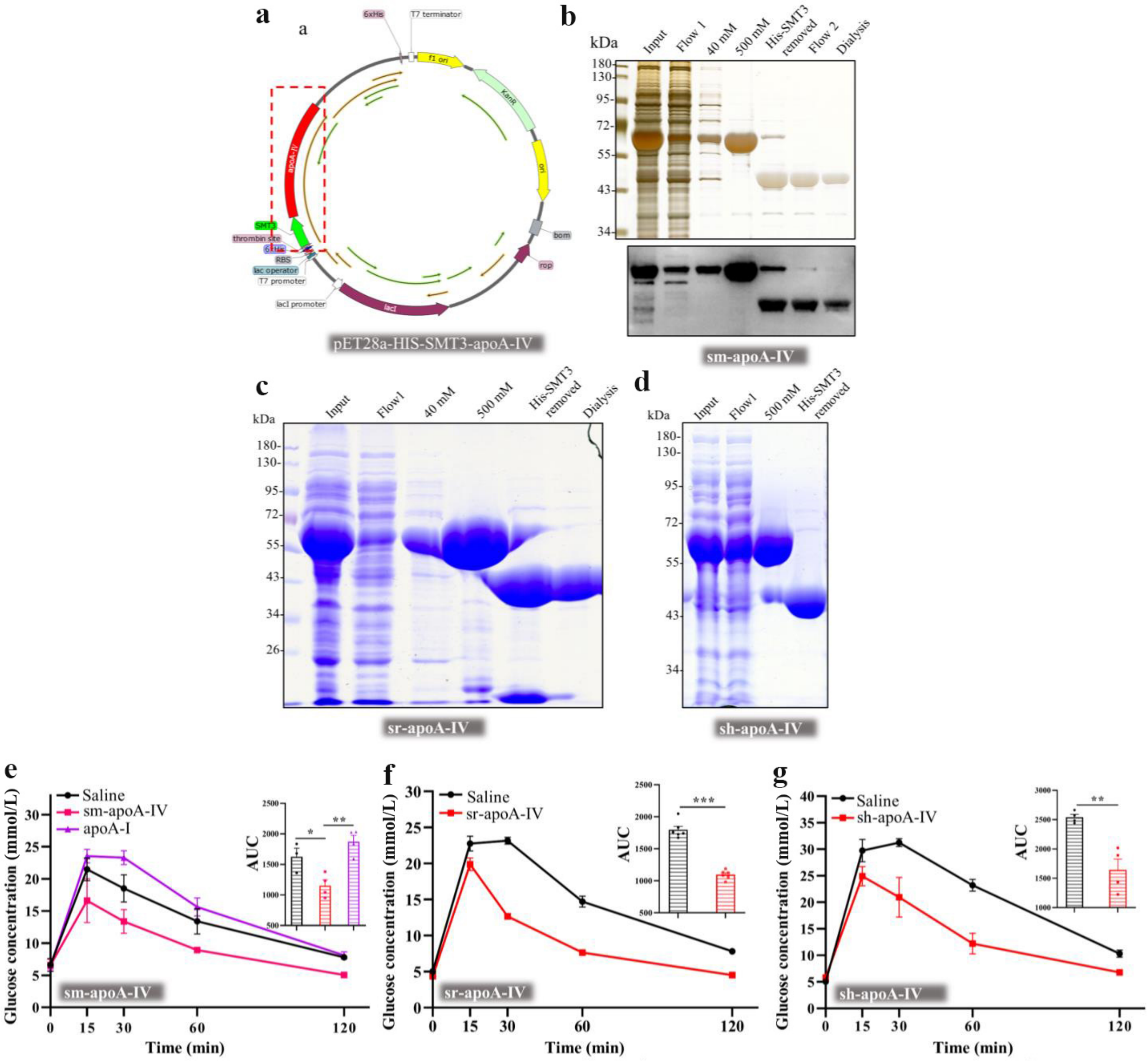

Supplementary Figure S4 Glucose tolerance is improved in WT mice with the administration of apoA-IV of different species. (a−d) Purification of different species of recombinant apoA-IV protein using a prokaryotic expression system. (a) Plasmid construct for recombinant apoA-IV protein expression system. The red dotted box represents the cloning site for insertion of apoA-IV nucleotide sequences of different species. (b) Purification of recombinant mouse apoA-IV. (c) Purification of recombinant rat apoA-IV. (d) Purification of recombinant human apoA-IV. (e) The ipGTT in WT mice (n = 3−4) with saline, sm-apoA-IV (1.5 mg/kg body weight) or apoA-I (1.5 mg/kg body weight) treatment. (f) The ipGTT in WT mice (n = 6) with saline or sr-apoA-IV (1.5 mg/kg body weight) treatment. (g) The ipGTT in WT mice (n = 4) with saline or sh-apoA-IV (1.5 mg/kg) treatment. Data are presented as mean ± SEM. Statistical significance was determined by the two-tailed Student's t-test. $^{*}P < 0.05$, $^{**}P < 0.01$, $^{***}P < 0.001$. ipGTT, intraperitoneal glucose tolerance test. AUC, area under the curve. sm-apoA-IV, signal peptide-removed mouse apoA-IV. sr-apoA-IV, signal peptide-removed rat apoA-IV. sh-apoA-IV, signal peptide-removed human apoA-IV.

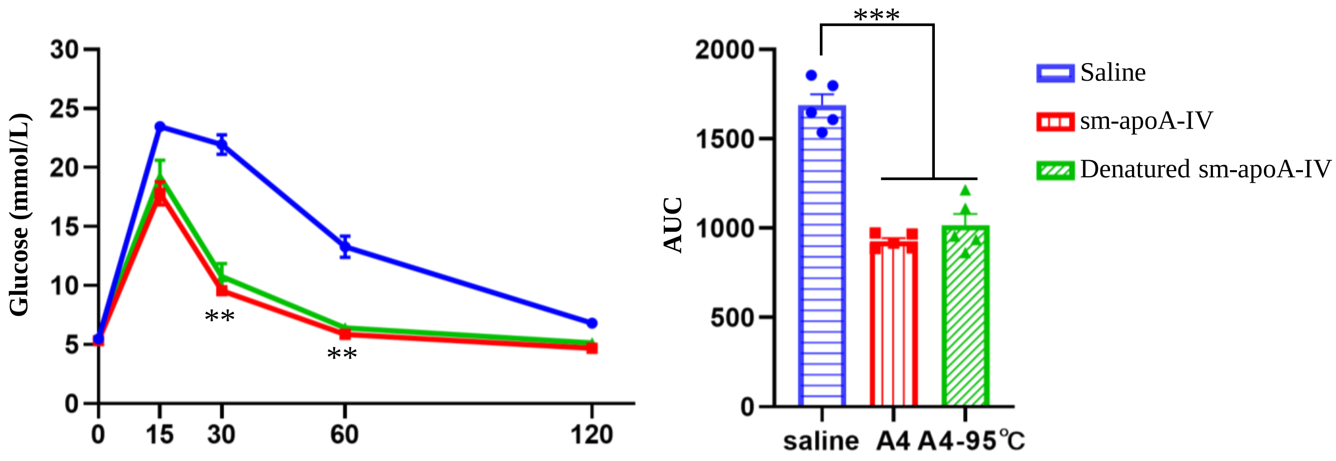

Supplementary Figure S5 Denatured apoA-IV improves glucose tolerance. The recombinant sm-apoA-IV was subjected to denaturation by boiling at 95℃ for 10 min. Equal amount (6 mg/kg body weight) of denatured sm-apoA-IV or non-denatured sm-apoA-IV was administrated to mice (*n* = 5) for ipGTT, respectively. Data are presented as mean ± SEM. Statistical significance was determined by the two-tailed Student's *t*-test. $^{**}P < 0.01$, $^{***}P < 0.001$. ipGTT, intraperitoneal glucose tolerance test. AUC, area under the curve. sm-apoA-IV, signal peptide-removed mouse apoA-IV.

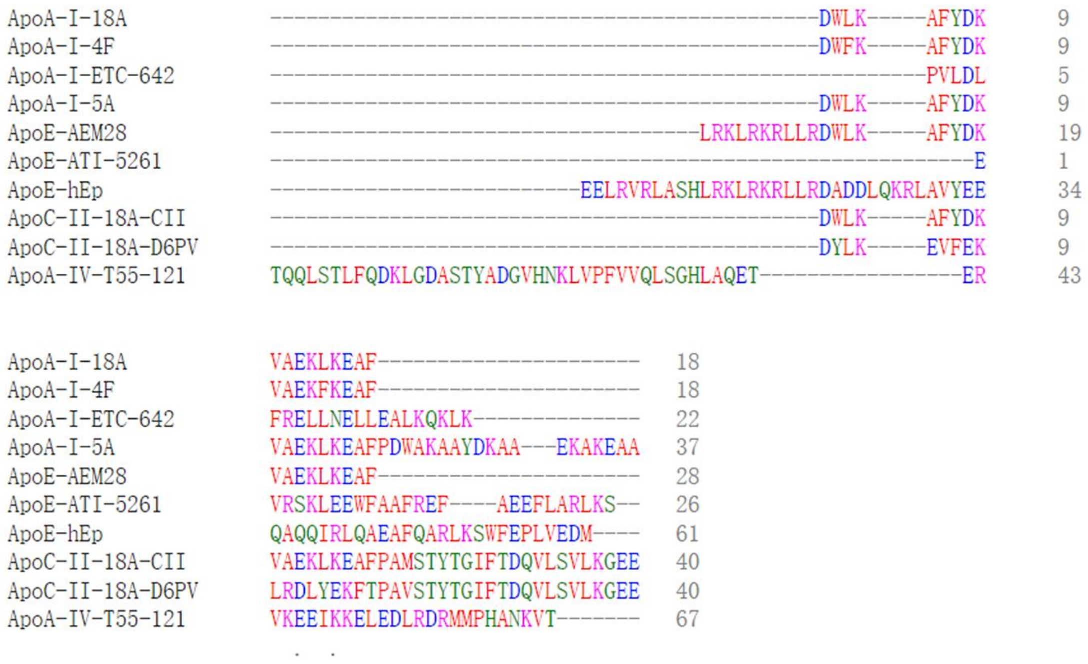

Supplementary Figure S6 The alignment of apoA-IV functional peptide T55−121 with other apolipoprotein mimetic peptides. ApoA-IV functional peptide T55−121 was aligned with other apolipoprotein mimetic peptides, including apoA-I, apoA-E, and apoC-II via Clustal Omega website.
